# Medical News

**Published:** 1853-03

**Authors:** 


### MEDICAL NEWS.

THE NEW YORK MEDICAL GAZETTE appears in a new and improved dress, and is henceforward to be published monthly. It has taken a firm hold of the Profession.

THE BRITISH AND FOREIGN MEDICO-CHIRURGICAL REVIEW has adopted the plan of affixing the *names of reviewers* to many of the reviews which appear in that Journal. The Journal, it should, however, be borne in mind, is published without the usual responsibility of editorial names, and it is not intended "to lay aside altogether the incognito" in reviews.

At a meeting of the College of Physicians of Philadelphia, held February 2d, 1853, the following gentlemen were elected Delegates to the American Medical Association: Isaac Hays, George Fox, Henry Bond, W. S. W. Ruschenberger, G. Emerson, J. Rodman Paul, Chas. Evans, William Ashmead, John Bell, Ed. Hallowell, Alfred Stillé, Joseph Carson, R. La Roche, Ed. Hartshorne, Robert Bridges.

NORTHERN MEDICAL ASSOCIATION.—At a meeting of the Northern Medical Association, held January 6, 1853, the following members were elected officers for the present year:

*President.*—Dr. Benjamin S. Janney.

*Vice-President.*—Dr. N. L. Hatfield.

*Counsellors.*—Dr. Joseph R. Bryan, John F. Lamb, Isaac Remington, Wm. Mayburry, and John Uhler, Jr.

*Secretary.*—Dr. J. Henry Smaltz.

*Corresponding Secretary.*—Dr. Isaac Remington.

*Reporting Secretaries.*—Drs. John Rhein and C. P. Turner.

*Delegates to the American Medical Association.*—Drs. L. Curtis, B. S. Janney, N. L. Hatfield, J. F. Lamb, R. J. Levis, and Isaac Remington.

The Naval Medical Examination Board met at Philadelphia on the 15th of December, and has reported the following Assistant Surgeons as qualified for promotion: 1, Wm. Lowber; 2, P. J. Horwitz; 3, B. Rush Mitchell; 4, D. E. Phillips; 5, Jas. Hamilton; 6, J. L. Burtt. Thirty-four candidates for admission into the Navy as Assistant Surgeons, were examined, and the following nine were selected as the best qualified: 1, James H. Stuart, of Pennsylvania; 2, J. Pembroke Thorn, of Virginia; 3, John M. Browne, of New Hampshire; 4, John T. Taylor, of Delaware; 5, Henry Clay Caldwell, of Virginia; 6, Thomas J. Turner, of Pennsylvania; 7, William T. Hord, of Kentucky; 8, Wentworth R. Richardson, of Massachusetts; 9, A. Clarkson Smith, of Pennsylvania.

The Medical Profession in Paris.—The Medical Directory of Paris, published by *L'Union Médicale*, gives the following numbers as to our Parisian brethren. Doctors of Medicine and of Surgery, 1337: officiers de santé (an inferior grade,) 179; pharmaciens, 423; midwives, 277. From the 1st of January, 1851, to 31st of December, 1852, there died in Paris 39 doctors of medicine; in the two previous years 64 had died. In the year just elapsed, 88 new practitioners set up in the capital. This year's list contains 15 medical men less than the last. The Directory also gives the numbers in the districts surrounding Paris, and from these statements it would appear that there is a great disproportion between doctors and patients. There are in fact less than 500 inhabitants for one medical man; and when it is considered how many of these apply to public institutions, very little is left for individual practitioners. *L'Union Médicale* warns young men from settling in Paris, as the exuberance of professional men is enormous.—*London Lancet.*

Death of Dr. George Gregory.—This distinguished author and physician died after a severe illness, on Wednesday, the 25th of January last, in London. He was well known to the Profession and to the public as the Physician to the Small-pox and Vaccination Hospital, as a Lecturer on the Practice of Medicine at St. Thomas's Hospital, and as the author of treatises on the theory and practice of medicine, and on eruptive fevers, as well as of several papers which have from time to time appeared in the "Transactions" of the Royal Medical and Chirurgical Society, and in other journals. His first work has been so highly appreciated, that it has not only gone through six editions in this country, but has been most favorably received in the United States of

America, and in our East Indian territories, and is still used as the text-book of most of the Army medical officers.—*London Med. Times and Gaz.*, Jan. 29, 1853.

Death of Sir William Newbigging.—We have to record the death of this highly respected surgeon, which took place at his residence in Edinburgh, on the 23d of October last. Mr. Newbigging was born at Lanark in 1772; and, after pursuing his studies at Edinburgh, became a Fellow of the Royal College of Surgeons of Edinburgh in 1799.

Sir William was a Fellow of the Royal Society of Edinburgh, was formerly President of the College of Surgeons, and Vice-President of the Wernerian Society. The interest which he took in the cultivation and advancement of useful knowledge led him, when his retirement from practice left him more at leisure, to become a fellow of the Scottish Society of Arts when he was in his 77th year.—*London Journ. Med. Science.*

Obituary—Mr. Alexander Walker, the ingenious author of a work on the "Nervous System," and others on "Beauty," "Woman," "Intermarriage," &c., which displayed much original research, died at an advanced age at Leith, on Tuesday, December 7th.—*London Lancet.*

Death of Jonathan Pereira, M. D., F. R. S., &c. In all ages and in all conditions of society it has been customary, on the death of any distinguished individual, to commemorate the name of the deceased by recording the good deeds which in life rendered him illustrious. This has been done, not so much, perhaps, as a tribute of respect to the dead, as an incentive to the practice of virtue by the living; and surely there is no character which presents so many opportunities for useful comment and example as that which, amidst the difficulties of circumstances, has triumphed over obscurity, and risen into honorable fame. This was the case with the late Dr. Pereira, whose determination of purpose and untiring industry enabled him to accomplish more in the the space of one short life than is usually effected in that of many longer ones.

The subject of our present memoir was born in the parish of Shoreditch, in London, on the 22d of May, 1804. He received the rudiments of his education at some of the small schools in the neighborhood; and when he was about ten years of age he was placed under the tuition of a classical master of no ordinary attainments. This gentleman kept an academy at No. 10 Queen street, Finsbury, and Pereira remained with him for a period of four years, during which time he obtained the friendship of his preceptor, who was accustomed to speak of him as a boy of considerable merit. When he left school he manifested a strong desire to enter the medical profession; and accordingly at the age of fifteen, or a little earlier, he was articled as an apprentice to Mr. Latham, an apothecary of the City-road. There he remained between two and three years, when his master became the subject of mental disease. This led to the breaking up of the practice and to the cancelling of Pereira's indentures. While he was with Mr. Latham he studied hard at his classics, and he drew a vocabulary of Latin terms for his guidance in dispensing.

At the close of the year 1821 he became a pupil at the General Dispensary in Aldersgate street, and he there attended the prelections of Dr. Clutterbuck on chemistry, materia medica, and practice of physic. He also availed himself of the lectures which were occasionally given by Dr. Birkbeck, on natural philosophy, and by Dr. Lambe, on botany. In the year following he entered to the surgical practice of Saint Bartholomew's Hospital. While thus engaged in the prosecution of his studies, a vacancy occurred in the office of apothecary at the Dispensary. This appointment he was anxious to secure for himself, but as he was not yet qualified for it, it was necessary that he should proceed at once to the hall, and obtain its license: this he did on the 6th of March, 1823, when he was only eighteen years of age. In the same month, he was appointed to the Dispensary, and we may date his illustrious career from that time. The salary at the Dispensary was not large—it was, in fact, only £120 per annum; and, with the view of increasing his income, he formed a class for private medical instruction. This he had but little difficulty in doing, as the lectures at the Dispensary were largely attended. His success in that undertaking was very great, and he thought it desirable to publish a few small books on the subjects in which he found his pupils most deficient. These were a translation of the "Pharmacopœia" for 1824, with the chemical decompositions; the "*Selecta è Prescriptis*;" a manual for the use of students; and a "General Table of Atomic Numbers, with an Introduction to the Atomic Theory." These works were published in the course of the years 1824–5, 6 and 7; they had a very extensive sale, and two of them are in existence at the present time.

In the year 1825 he passed the College of Surgeons, and in the year following he succeded Dr. Clutterbuck as a lecturer on chemistry. At that time he was only twenty-two years of age, but his appearance was commanding, and he therefore looked much older. His first lecture was given to a large class of pupils and friends. It was eminently successful, and he received the warm congratulations of his numerous admirers. Then, as ever afterwards, he sought to dazzle by the novelty of his facts and the profusion of his illustrations. His lecture-table was covered with specimens, and, among other things, he exhibited the new element, bromine, which Bolard, of Montpelier, had just then discovered.

In the course of a year or two after that time, he began to collect the facts for his "Materia Medica." He saw that the whole subject of pharmacology was involved in the greatest confusion, that its principles were misapprehended, and that its doctrines were founded in absurdity and conjecture. From this chaos and darkness he determined to relieve it. Accordingly, he commenced a diligent search for all the facts of the science; he studied the ancient fathers of physic, and made himself master of the literature of the subject, from the earliest period of history; he collected the works of English writers, and he undertook the study of French and German, in order that he might read those of the Continent. At that time he devoted his whole energies to the subject, and worked for about sixteen hours a-day. He was accustomed to rise at six in the morning, and to read, with but little interruption, until twelve at night. This he continued to do for several years; and had he not been possessed of an iron constitution, of great physical endurance, and of a most determined purpose, he would unquestionably have sunk under it. As it was, the closeness of his application occasioned several slight attacks of epilepsy, and a frequent determination of blood to the head. After a short time he began to give lectures on materia medica, as well as on chemistry, at the Dispensary; and he must have been so completely engaged that he could not find leisure for original investigation. In fact, from the year 1827 until that of 1835, his name appears but once in the journals of the time. This occurred in 1829, when he published a short paper in the *London Medical and Physical Journal*, on the adulteration of hydriodate of potash.

In the year 1832 his affairs were in so prosperous a state that he ventured to leave the Dispensary, and to get married. He resigned his appointment in favor of his brother, and he commenced practice as a surgeon in Aldersgate street. A reference to THE LANCET of the time will show that he left the Dispensary about twelve months before the notorious quarrel occurred at that Institution. It is probable that he foersaw the approaching storm, and retired from the falling house; but be that as it may, he had, in the year of his marriage, joined the new medical school in Aldersgate street; and in the year following he was elected to the Chair of Chemistry in the London Hospital. For a period of six years he lectured at both of these places on three subjects —namely, on Chemistry, Botany and Materia Medica; and during the whole of each winter session he was accustomed to give two lectures daily. While he was at the Aldersgate Medical School he became very intimate with Dr. Cummin, who was then the editor of the late *Medical Gazette*; and in consequence of this and also of his great popularity as a teacher, he was engaged to publish his lectures on Materia Medica in that journal. They extended over a period of two years, viz., from 1835 to 1837, and amounted to seventy-four in number. There can be no doubt that they greatly added to his reputation; for we find that they were translated into the German, and re-published in India. In 1839, he re-produced them in another form—viz., in his "Elements of Materia Medica," and this work was so much appreciated that the whole of the first part was bought up long before the second was ready for delivery. A second edition was therefore immediately called for, and it appeared in the year 1842. Before this date, however, viz. in 1839, he had been chosen Examiner in Materia Medica in the University of London; and in 1841 he had been elected Assistant-Physician to the London Hospital He took his degree at Erlangen, in 1840, and he obtained his license at the College of Physicians directly afterwards. About the same time he was invited by some of the authorities of St. Bartholomew's Hospital to lecture at the medical school of that Institution, and the arrangements for his so doing had been almost completed, for a syllabus of the course was actually published; but when it was notified to him that he would be required to give up his other appointments, he refused to relinquish his position at the London Hospital, at which institution he had experienced great kindness. He immediately afterwards, however, gave up the Aldersgate School.

In 1842, he gave two short courses of lectures at the rooms of the Pharmaceutical Society, and, in the year following, he was appointed its first professor. During that year he published his work on Food and Diet, and he was elected on the Council of the Royal Society. By that time, his practice as a physician had become rather extensive, and as it was rapidly increasing, he determined to throw aside his more scientific pursuits. Accordingly, in 1844, he resigned a part of the course of chemistry at the London Hospital into the hands of Dr. Letheby; in 1845 he gave up a larger portion of it; and in 1846 he relinquished it altogether. He continued, however, to lecture on Materia Medica at both the hospital and the Pharmaceutical Society, and there is no reason for believing that he contemplated any change in this matter until the new regulations of the Apothecaries' Society transferred his course to the summer session. This arrangement interfered with his usual habits, and also with his ideas of the importance of the subject, and consequently, in 1850, he resigned his lectureship at the hospital, though he still continued to deliver a winter course at the Pharmaceutical Society. In 1845, he was elected a fellow of the College, and, in 1851, he became a full physician at the hospital. He had now reached the summit of his ambition: his reputation as an author was established, and the rewards of industry were falling thick about him. He was a fellow of many scientific societies, he was in constant communication with the learned of all countries, he was intimately connected with many of the greatest institutions of the metropolis, and was, in fact, their brightest ornament; he had collected around him a large circle of friends and admirers, and he saw before him the prospect of wealth and happiness. In the midst of all this, however, he was stricken down, and that so suddenly, that he had hardly time to take leave of those who were about him. He died on the 20th of January last, and within one week of health and hope he was placed in his last resting place. His funeral took place at Kensal-green on the Thursday following, in the presence of a large number of friends and pupils, who mourned in silent grief.

A retrospect of the labors of this distinguished physician will show that he was a man of no ordinary capacity, that he was characterized by qualities which always ensure success. He had an unquenchable thirst for knowledge, an indefatigable spirit, unbounded industry, and a determination of purpose that was irresistible. Whatsoever he did he did well, and he therefore made his performances as valuable to others as they were creditable to himself. This is evidenced by the works to which we have alluded, for they have made for him the reputation which he possessed, and have given to others the means of acquiring knowledge which is not to be found elsewhere. The great peculiarity of his efforts is, that he aimed more at bringing within our reach the treasures of other men's minds than of exposing those of his own. He has, indeed, been charged with a want of originality, and, most certainly, if we estimate him by the value of his own independent researches, he is open to such a charge; but it must also be admitted that it is an equally useful element of the human mind, that faculty which urges men to gather up the scattered facts of science, and to mould them into a shape that may be made available to all. This has our author accomplished, and the result of his labors will endure beyond the efforts of originality, for they will last through all time, as a monument of his industry.—*London Lancet,* Feb. 1853.

Death from the administration of chloroform.—A person named Henry Hollingworth, a factory operative from Newton-moor, near Hyde, has fallen a victim, at the Manchester Royal Infirmary, to the use of chloroform, administered to nullify the pain consequent upon a severe operation. An inquest was held on view of the body by Mr. Herford, coroner for the borough, when the following evidence was given:

Mr. John Wright Baker, house-surgeon at the Royal Infirmary, said the deceased was admitted on the 16th Dec., on account of a malignant tumor on the right thigh, to remove which an operation was performed, as it was looked upon as a cancerous tumor, though enveloped in much doubt, as those tumors often are. He was in a bad state of health when admitted, and everything was done to improve his health previous to the operation. A consultation, as I understood, had been held previous to his admission, and it had then been determined that the operation should be performed, of course with the consent of the patient. The consultation was of all the medical men of the infirmary. The deceased, the day before the operation, said he was ready for it, but wished to have chloroform. Almost every patient takes it. I said he should have it if he wished it, and that he would feel no pain, and that I would do all I could to support his strenth. I did not give him any caution. We have given chloroform frequently, and never had a fatal case before. At eleven o'clock on Friday, the 24th Dec., the operation took place. There were present Mr. Jordan, as the operator, Mr. Beever, as his assistant in the operation, Mr. Wilson, Dr. Renaud, and Dr. Wilkinson (all members of the honorary medical staff of the infirmary,) and myself. Mr. Frederick Heath, a qualified surgeon, administered the chloroform. The man was very much excited, struggled, and talked fast. The chloroform was administered slowly, and every precaution was taken to prevent any danger; and the medical men remarked two or three times how very long it was in taking effect. He at last became insensible in about seven minutes at least. Mr. Jordan commenced the operation by an incision into the skin covering the tumor. I was assisting the surgeon when Mr. Heath directed my attention to the patient's face. This was about five minutes after the operation had commenced. I then observed congestion about the face, but there was no stertorous breathing. His pupils appeared almost to have ceased to act. His breathing was become exceedingly slow, and he seemed to be sinking fast. I directed the attention of the operator and other medical men to these symptoms. The operation was then suspended, and means resorted to for restoring animation, but the pupils had ceased to act, and had become fixed almost immediately. He gave one strong gasp, and then to all appearance was dead. In administering the chloroform, successive doses were given until it took effect. Every dose consisted of a drachm, taken at intervals in an inhaler. Constitutions differ with regard to the effect produced by chloroform, but we use every precaution to prevent injury; and I am satisfied that the surgeons did their duty in the administration of the chloroform and in the operation.

Mr. Jordan was examined, and stated, in corroboration of Mr. Baker's evidence, that more time elapsed than usual before insensibility was produced, and then it was not complete, for after the incision was made, the man, more than once, said a cat was scratching him. Chloroform was generally administered in cases of operation, unless there were circumstances which, in the opinion of the surgeons, rendered it undesirable. Mr. Heath was a competent person to administer chloroform. The post-mortem examination showed that asphyxia, caused by chloroform, produced the death. There was a congestion both of the brain and lungs.

Verdict—"Died from the effects of chloroform."

It is, we understand, the determination of the medical staff in all cases requiring the use of this deadly though valuable agent, to have one person to administer it, and another to scrutinize the effects upon the patients, in order to avoid a second fatality of the kind.—*London Lancet.*

THE FOUNDER OF THE BRITISH MUSEUM.—Sir Hans Sloane was born, according to some documents in the library of his splendid collection at the British Museum, in the County Down, Ireland, at a place called Killeagh, April 16, 1660. He stated in his will that the collection he was bequeathing the nation was richly worth £80,000. It contained 200 volumes of dried plants in the form of a *hortus siccus*, 30,000 mineral and other specimens of great interest in natural history, with a library of 50,000 volumes, and 3566 very rare manuscripts. There are two pictures of this great and talented Irishman in the Museum. One would like to see them more generally known. It appears, from the little written of Sir Hans Sloane, that it was in Ulster, in Ireland, he first imbibed that love of scientific pursuits that has since rendered his name, and we fear only his name, illustrious: in France and Ireland, in fact, was laid the foundation of his great museum. Like Hunter, Mozart, Goldsmith, Haydn, Johnson, and a legion of other great men, we find Sloane in early years struggling a good deal with adversity. Before he was of age he had several severe attacks of hæmoptysis, which threatened him with an early grave, like Laennec, Bichât and others; death, however, spared him till he had done his work. Stahl, Ray, and another great countryman, Robert Boyle, were then in the ascendant, all of whom were known to Sloane, and helped to form his mind. And yet who thinks, amid the winged bulls from Nineveh, and the magnificent collection crowded at present in the huge building of the British Museum, of Sir Hans Sloane or Boyle. We think of Watt whenever we see a steam engine, because with one happy thought he has made it the last wonder of the world in a utilitarian point of view; the great workers in the mine of abstract science and philosophy we disregard. In 1683, young Sloane set off for France, and there seems to have been delighted with the botanical collections and lectures of Tournefort, and spent a year collecting plants for the museum. We find him next going out to the West Indies, a young man, under 30, physician to the Duke of Albemarle, and in spite of many crosses and disappointments, still further adding to his specimens 800 species of rare and valuable tropical plants. These two collections form the first nucleus of the British Museum. It seems all this time he never forgot his native country, Ireland, and we should in all probability have those specimens now in Trinity College, Dublin, and the British Museum (which would be a great blessing) quite a different institution, but that Sloane got married to a very rich wife, and settled permanently in London. In 1693, he became Secretary of the Royal Society, and in 1727, was appointed physician to the king, and succeeded the great Newton as president of the Royal Society. George I. made him a baronet, and he died, at the age of 93, in 1753.—*Dublin Medical Press.*

Five Cases of Persons struck by Lightning.—In five men who suffered from this accident, M. Minourid observed burns presenting black eschars, with destruction of the epidermis, rete mucosum, and of a part or the entire thickness of the dermis, covered with a dark-colored phlyctenæ, surrounded by an erythematous redness; the eschars were large, irregular, ill-circumscribed, oval, or round, separated from each other by spaces of inflamed skin; no part of the body escaped, but they were particularly well marked on the outside of the lower extremities. It is remarkable that nowhere did the burn extend deeper than the dermis (the burn of the fourth degree of Dupuytren.) Would it appear, then, that the subcutaneous cellular tissue is a non-conductor of electricity? Of ordinary burns, those which most resemble the effects of lightning are caused by gunpowder explosions; but the latter differ essentially, by bearing the marks of the grains of powder in the skin. In one of these men who died instantly, the writer observed that, though it was then the month of December, and the body had been left during the night merely covered with a cloth, and exposed to the air, yet after a period of thirteen hours, it was yet warm.—*Annali Universali di Medicina.*

